# 

**Published:** 1888-03

**Authors:** 


### CONTENTS, MARCH, 1888 (VOL. III, NO. IX).

ORIGINAL ARTICLES.—The Treatment of Acute Dysentery; J. W. McLaughlin, M. D., Austin, Texas .................. 361
Antipyrine as a Hæmostatic; W. M. Powell, M. D., Albany, Texas .................................................. 366
Chronic Granular Conjunctivitis, or Trachoma; F. E. Yoakum, M. D., Greenville, Texas .................... 375
Etiology and Treatment of Talipes, Varus and Vulgus, congenital and acquired; C. McReynolds, M. D., Fort Smith, Arkansas .................................................. 369
Cocaine in Cervical Dilatations for Dysmenorrhœa; M. J. Birdsong, M. D.; Greenville, Texas ...................... 375
Little Things in Surgery; J. E. Roach, M. D., Sipe Springs, Texas .................................................. 376
CORRESPONDENCE.—Veratrum in Pneumonia; letter from Dr. Hutchings .................................................. 378
Taxation Without Representation; letter from Dr. Talley .. 379
Proposed Changes in the Physicians' Mutual Benefit Association of Texas; letter from Dr. Vellum .............. 380
Proposed Changes in the Physicians' Mutual Benefit Association; letter from Dr. S. A. Stone .................. 383
A Second Sarah; Return of Menses in Old Age and Second Sight; letters from Dr. Knox ............................ 381
A Lusus Naturæ; letter from Dr. Mendenhall ............ 382
SOCIETY NOTES.—East Texas Medical Association ........... 384
Third Annual Meeting Lone Star Medical Association ..... 385
Physicians' Mutual Benefit Association; List of those who paid Assessment No. 5; Mrs. Starley's Receipt; Statement of Secretary; A Stirring Appeal to the Profession in Behalf of the Order, by Dr. Day. President Louisiana P. M. B. A.; Constitution and By-Laws ............ 385–390
CULLINGS FROM CONTEMPORARIES .................... 392–403
EDITORIAL.—Taxation Without Representation .............. 404
MEDICAL NEWS AND MISCELLANY ............................. 405
NECROLOGICAL .................................................. 407
ADVERTISER'S NOTICES .......................................... 408

